# Cell Membrane-Targeted Antibacterial Synergy of Citric Acid–Sodium Hypochlorite Against *Salmonella* Typhimurium on Cherry Tomatoes

**DOI:** 10.3390/foods14193390

**Published:** 2025-09-30

**Authors:** Tianyu Yin, Zhan Huang, Xinhui Zhang, Jin Huang, Zhehao Yang, Qiao He, Mingming Guo

**Affiliations:** National-Local Joint Engineering Laboratory of Intelligent Food Technology and Equipment, Fuli Institute of Food Science, Zhejiang Key Laboratory for Agro-Food Processing, Zhejiang International Scientific and Technological Cooperation Base of Health Food Manufacturing and Quality Control, College of Biosystems Engineering and Food Science, Zhejiang University, Hangzhou 310058, China; 22313079@zju.edu.cn (T.Y.); 22413039@zju.edu.cn (Z.H.); 12213076@zju.edu.cn (X.Z.); 12413056@zju.edu.cn (J.H.); 12313042@zju.edu.cn (Z.Y.); heqiao@zju.edu.cn (Q.H.)

**Keywords:** organic acids, washing sanitizers, synergistic sanitizing efficacy, *Salmonella* typhimurium, antibacterial mechanism

## Abstract

Foodborne illness outbreaks from fresh produce underscore the urgent demand for sanitizing strategies that ensure safety while minimizing harmful by-products from high-dose chemical disinfectants such as sodium hypochlorite (NaOCl). Low-concentration combinations of organic acids and washing sanitizers were systematically evaluated to identify synergistic antibacterial effects, and citric acid (CA) was found to markedly potentiate the activity of NaOCl against *Salmonella* Typhimurium through a sequential assault on the cell envelope. A low-dose combination of sub-inhibitory concentrations (1/2 MIC of CA and 1/4 MIC of NaOCl) exhibited robust synergy, achieving a >6 log CFU/cm^2^ reduction in the pathogen on a cherry tomato model within 3 min. Moreover, this synergistic entry leads to profound disruption of membrane integrity, resulting in leakage of nucleic acids and proteins, extensive oxidative damage, hyperpolarization, and cell lysis, as confirmed by electron and confocal microscopy together with physicochemical assays. Mechanistic investigation revealed that oxidative damage from NaOCl amplified CA-induced membrane acidification and permeability, facilitating deeper sanitizer penetration and accelerating envelope destruction. Collectively, these findings uncover a membrane-targeted synergistic mechanism, providing a solid scientific basis for the development of novel, low-residue, and high-efficacy food safety interventions.

## 1. Introduction

Fresh produce is susceptible to microbial contamination during cultivation and post-harvest processing, and its consumption when raw makes it a major source of foodborne illness worldwide, resulting in substantial public health and economic burdens [1,2]. Cherry tomatoes are a cornerstone of a healthy diet, yet their consumption carries a risk of foodborne illness due to contamination by pathogens such as *Salmonella* Typhimurium and Shiga toxin-producing *Escherichia coli*, facilitated by their elevated surface pH and direct soil contact during cultivation [3,4].

*Salmonella enterica*, especially non-typhoidal serovars, poses a persistent challenge due to its ability to adhere to and survive on plant surfaces and internal tissues [5]. In the United States, non-typhoidal *Salmonella* remains the leading bacterial cause of foodborne illness, with over one million cases annually [6]. Data from Interagency Food Safety Analytics Collaboration indicate that fruits and seeded vegetables—represented by tomatoes—account for 26.7% of *Salmonella*-related foodborne illnesses [7]. *Salmonella* Typhimurium, a predominant serovar, is frequently isolated from contaminated vegetables [8] and is responsible for 29.1% of *Salmonella*-positive cases in fresh produce globally [9]. It has been implicated in multiple outbreaks, such as a 2021 incident linked to salad greens that resulted in 31 illnesses [10]. *Escherichia coli* O157:H7 (*E. coli* O157:H7) poses a significant public health risk due to its extremely low infectious dose. It also survives in natural water sources, which facilitates transmission [11].

While chemical disinfection is essential for microbial safety, the growing consumer demand for minimally processed foods and heightened awareness of chemical residues have created an urgent need for strategies that are both effective and sustainable. Sodium hypochlorite (NaOCl) remains the most widely used disinfectant in the produce industry due to its broad spectrum of activity and affordability. However, its efficacy is highly concentration-dependent, which can lead to the formation of carcinogenic disinfection by-products, such as trihalomethanes and chlorite residues [12], and harmful aerosols can be produced during washing, posing health risks to workers [13]. Moreover, high organic loads in recirculated wash water reduce chlorine efficacy and increase the risk of cross-contamination [14]. To mitigate these risks, it is recommended that NaOCl be maintained below 200 ppm and to rinse produce thoroughly with clean water after disinfection [15]. Peracetic acid (PAA) has been proposed as an alternative to chlorine-based sanitizers due to its strong and broad-spectrum antibacterial activity, rapid action, and environmentally friendly decomposition into acetic acid, water, and oxygen [16,17], making it suitable for reducing cross-contamination in food processing environments. Nevertheless, its strong oxidative potential may impair produce surface integrity and degrade sensitive nutrients such as vitamin C, thereby reducing nutritional quality [18]. Consequently, optimizing sanitizer use or exploring alternatives is crucial to enhance efficacy while reducing chemical input.

Organic acids, such as citric acid (CA) and tartaric acid (TA), are considered safer alternatives, with Generally Recognized as Safe (GRAS) status, and are widely used for antibacterial purposes in food preservation. Their antibacterial activity primarily involves the disruption of bacterial membrane integrity and interference with the intracellular metabolism [19]. However, their bactericidal activity when used alone is often limited, requiring concentrations that can compromise product quality. This dilemma has spurred interest in synergistic antibacterial strategies. For example, 2% malic acid combined with electrolyzed water achieved a 99.9% reduction in *Listeria* on food-contact surfaces, likely due to enhanced membrane permeability [20]. Although combining disinfectants to enhance efficacy is not a new concept, a significant knowledge gap persists: there has been a lack of systematic investigation into the synergy between food-grade organic acids and conventional sanitizers like NaOCl, particularly regarding dose optimization to minimize chemical inputs. Furthermore, the underlying cellular mechanisms driving such potential synergies remain poorly understood, hindering the rational design of highly effective, low-residue disinfection protocols.

In this study, combinations of organic acids (CA and TA) with common food sanitizers (NaOCl and PAA) were screened and optimized to identify formulations with enhanced antimicrobial synergy. Their bactericidal efficacy was evaluated both in vitro and on a cherry tomato model. After screening various formulations, this study focused on a promising formulation and investigated its membrane-targeted antibacterial mechanisms by examining membrane integrity, oxidative stress, and macromolecular damage. These results provide mechanistic insights for optimizing disinfection strategies in fresh produce processing and highlight the potential of organic acid-sanitizer synergies for improving food safety.

## 2. Materials and Methods

### 2.1. Materials

Organic acids (CA, TA) and washing sanitizers (NaOCl, PAA) were purchased from Sinopharm Group Chemical Reagent Co., Ltd. (Shanghai, China). The NaOCl solution contained 6–14% chlorine; experimentally, the available chlorine content was titrated to 7.6% using the sodium thiosulfate standard titration method under acidic conditions. All combinations in this study were prepared based on this experimentally determined available chlorine concentration to ensure consistency and comparability across formulations. The PAA was prepared following the manufacturer’s instructions, by mixing equal volumes of two stock solutions: Solution A, consisting of glacial acetic acid and sulfuric acid, and Solution B, containing hydrogen peroxide. The solutions were mixed in a glass container at a 1:1 ratio (*v*/*v*) and allowed to react for 24 h at room temperature before use. The final concentration of PAA was determined to be 0.19 g/mL using potassium permanganate standard titration followed by the indirect iodometric method.

*Salmonella* Typhimurium (ATCC 14028) and *E. coli* O157:H7 (ATCC 35150) were obtained from the Guangdong Microbial Culture Collection Center and stored at −80 °C upon arrival. For isolation, *Salmonella* Typhimurium was streaked on xylose lysine deoxycholate (XLD) agar and *E. coli* O157:H7 on eosin methylene blue (EMB) agar, both of which are selective media for the respective strains. All strains were cultured in nutrient broth (NB) medium to prepare bacterial suspensions. Bacterial concentration was measured at 600 nm and referenced to a previously established OD_600_–CFU calibration curve, where OD_600_ > 1.0 corresponds to ~9 log CFU/mL. The suspension was adjusted accordingly. After centrifugation and washing, the bacteria was resuspended in 0.85% physiological saline before use.

### 2.2. Determination of Antibacterial Effect of Organic Acids and Washing Sanitizers

According to the guidelines approved by the National Committee for Clinical Laboratory Standards (NCCLS), the minimum inhibitory concentrations (MIC) and minimum bactericidal concentrations (MBC) of CA, TA, NaOCl and PAA against *Salmonella* Typhimurium and *E. coli* O157:H7 were determined. The method was also slightly modified based on the broth microdilution procedure [21]. A two-fold serial dilution method was employed to determine the MIC. The bacterial suspension was adjusted to 6 log CFU/mL, and 100 μL of nutrient broth and 100 μL of the sample solution were sequentially added to a 96-well microplate to perform the dilution, followed by the addition of an equal volume of the bacterial suspension. After incubation at 37 °C for 18 h, the optical density at 600 nm (OD_600_) was measured using a microplate reader, with each treatment conducted in triplicate. Preliminary MIC values for the four solvents were obtained, and to achieve more accurate MICs, the experiment was conducted with different concentration gradients within a narrow range. The lowest concentration at which the turbidity in the 96-well plate remained below an OD of 1.0 and within ±0.2 of the control was considered the MIC. From the MIC-determined solution, 100 μL was spread on plate count agar (PCA) and incubated overnight at 37 °C. The MBC was defined as the lowest concentration resulting in no colony growth on PCA.

### 2.3. Evaluation of Synergistic Antibacterial Effects

#### 2.3.1. Screening for Synergistic Combinations

An MIC checkerboard method using a 96-well plate was employed for the combined antibacterial test. Different concentrations and ratios (1/8 to 1 MIC) of CA, TA, NaOCl and PAA were prepared. In well A1, 100 μL of broth was added as a blank control. In other wells, a single variable method was used to add 100 μL of different concentration gradients of the combined mixtures, followed by the addition of 100 μL of bacterial suspension, resulting in a final bacterial concentration of approximately 6 log CFU/mL per well. The 96-well plate was incubated at 37 °C for 18 h. MICs determined for the combinations of organic acids and sanitizers were used to calculate the FICI. In parallel, the pH of combination treatments was determined using a pH meter (Early Environmental Technology Co., Ltd., Shanghai, China).

The combined antibacterial effect was evaluated using the following formula [22]:

$$\mathrm{FICI}={\mathrm{FIC}}_{\mathrm{Organic}\;\mathrm{acid}}{(\mathrm{FIC}}_{O})+{\mathrm{FIC}}_{\mathrm{Washing}\;\mathrm{sanitiser}}\;{(\mathrm{FIC}}_{W})$$

where ${\mathrm{FIC}}_{O}={\mathrm{MIC}}_{O}\;\mathrm{in}\;\mathrm{combination}/{\mathrm{MIC}}_{O}\;\mathrm{alone}$, and ${\mathrm{FIC}}_{W}={\mathrm{MIC}}_{W}\mathrm{in}\;\mathrm{combination}/{\mathrm{MIC}}_{W}\;\mathrm{alone}$.

The MIC alone refers to the MIC value of single organic acid or washing sanitizer, respectively, and MIC in combination is the isoeffective concentration of organic acid or washing sanitizer in the combinations, respectively.

#### 2.3.2. Time-Killing Assays

A plate colony counting method was used to conduct four time-killing experiments on organic acids and washing sanitizers that showed favorable synergistic effects against *Salmonella* Typhimurium and *E. coli* O157:H7 [23]. The logarithmic phase bacterial suspension with a final concentration of about 6 log CFU/mL was inoculated into the NB containing the four combined mixtures; 0.85% physiological saline solution was used as the control group. The cultures were incubated at 37 °C for 0, 2, 4, 6, 12, and 24 h. At regular intervals, the solution was serially diluted to appropriate concentrations and 100 μL was spread on agar plates. After incubation at 37 °C for 24 h, the colonies were counted, and the relationship between the average number of colonies (log CFU/mL) and time was plotted.

### 2.4. Pathogen Reduction Efficacy During Cherry Tomato Washing

Cherry tomatoes were wiped with 75% ethanol to remove surface wax, then rinsed with sterile deionized water and placed under a laminar flow hood for UV sterilization for 2 h to eliminate the presence of natural microbiota on the fruit skin. *Salmonella* Typhimurium and *E. coli* O157:H7 solutions were mixed evenly in a 1:1 (*v*/*v*) ratio. A 1.0 cm × 1.0 cm area on the fruit surface was marked with a marker. Then, 50 μL of bacterial suspension was evenly applied to five points on the marked slice, and the inoculated fruit was left at room temperature for 2 h to allow pathogen attachment [24]. The two best combinations against mixed pathogen, the CA-NaOCl combination (1/2 MIC CA + 1/4 MIC NaOCl) and the CA-PAA combination (1/3 MIC CA + 1/3 MIC PAA), were prepared separately for control and sample groups. After 3 min of washing and air-drying, a 1.0 cm^2^ marked area was excised, placed in 10 mL of physiological saline, homogenized, serially diluted, and plated on agar for enumeration (CFU/cm^2^). Pathogen residues on the fruit surface were quantified after each treatment in the sample group.

### 2.5. Scanning Electron Microscopy Analysis

To further investigate the antibacterial mechanism of the selected formulation, structural damage to *Salmonella* Typhimurium was examined using scanning electron microscopy (SEM). Based on the observed bactericidal efficacy, the optimal CA-NaOCl combination was selected for treating *Salmonella* Typhimurium cells. Solutions for different treatments were prepared as follows: CA (1/2 MIC), NaOCl (1/4 MIC), and CA-NaOCl combination. Following a 3 min treatment, 1 mL of the sample was centrifuged at 8000 rpm for 5 min, and the pellet was resuspended in 1 mL of 2.5% glutaraldehyde solution to preserve cellular structures. The sample was subsequently washed with 0.1 M PBS (pH 7.4), fixed with 1% osmium tetroxide to stabilize lipids, and dehydrated through a graded ethanol series to ensure the complete removal of water. After critical point drying, the sample was coated with a conductive material and visualized under SEM (SU-8010, Hitachi Ltd., Tokyo, Japan) at magnifications of 20 k and 40 k to evaluate the ultrastructural changes.

### 2.6. Assessment of Cell Integrity

#### 2.6.1. Intracellular Nucleic Acid Release

A bacterial suspension was prepared to achieve a final concentration of 6 log CFU/mL, ensuring a uniform cell density for all treatments. Solutions for different conditions were prepared as follows: CA (1/2 MIC), NaOCl (1/4 MIC), CA (MIC), NaOCl (MIC), and CA-NaOCl combination. After mixing with the bacterial suspension for 3 min under controlled conditions, the samples were centrifuged at 8000× *g* for 3 min at 4 °C to remove cell debris, followed by washing twice with sterile physiological saline to minimize residual contaminants. The supernatants were collected and transferred to sterile tubes, and the nucleic acid release was quantified by measuring the absorbance at OD_260_ using a UV-visible spectrophotometer (UV-2600, Shimadzu Corporation, Fukuoka, Japan). This approach ensured the accurate detection of intracellular components released during membrane disruption [25].

#### 2.6.2. Intracellular Protein Release

The leakage of proteins was evaluated based on OD_280_ values, following the modified protocol reported by [26], with slight adaptations to suit the CA-NaOCl combination system. Following treatment under the same experimental conditions, cell suspensions from the treated and control groups were centrifuged at 8000× *g* for 3 min at 4 °C to separate the supernatants. The samples were subsequently washed to remove residual extracellular material. Protein content in the collected supernatants was determined by measuring absorbance at 280 nm using a UV–visible spectrophotometer. A standard curve was constructed using bovine serum albumin (BSA) to quantify the released proteins, reflecting the extent of membrane damage and intracellular protein leakage.

#### 2.6.3. Propidium Iodide Fluorescence Staining Analysis

Propidium iodide (PI), a membrane-impermeable fluorescent dye commonly used to assess bacterial membrane integrity [27], was used in this study. After centrifuging the treated and control cell suspensions to collect the supernatant and measuring the absorbance, the OD_600_ was standardized. The cells were mixed with PI to a final concentration of 10 μM and incubated in the dark at 37 °C for 30 min. Fluorescence intensity was monitored using a fluorescence spectrophotometer (Infinite E Plex, Tecan Austria GmbH, Grodig, Austria) with an excitation wavelength of 535 nm and an emission wavelength of 617 nm.

### 2.7. Cell Membrane Permeability Assay

#### 2.7.1. Outer Membrane Permeability

Bacterial suspensions were diluted to 6 log CFU/mL and treated with blank control (CK), CA (1/2 MIC), NaOCl (1/4 MIC), CA (MIC), NaOCl (MIC) and CA-NaOCl combination for 3 min. After elution, the solutions were mixed with a final concentration of 10 µM N-phenyl-1-naphthylamine (NPN) solution and incubated in the dark for approximately 30 min. The fluorescence intensity of each mixture was monitored using a fluorescence spectrophotometer (Infinite E Plex, Tecan Austria GmbH, Grodig, Austria) in a black 96-well plate, with an excitation wavelength of 350 nm and an emission wavelength of 420 nm. Maximum values were recorded.

#### 2.7.2. Inner Membrane Permeability

*Salmonella* Typhimurium suspensions were cultured in LB broth with 2% lactose, rinsed, and resuspended. The bacterial suspensions were mixed with different sample solutions and treated for 3 min. Samples were thoroughly mixed with a final concentration of 5 µM ortho-Nitrophenyl-β-galactoside (ONPG) solution and incubated at 37 °C with 150 rpm for 30 min [28]. Absorbance at 420 nm was read using a Multiskan GO microplate reader (Thermo Fisher Scientific, Waltham, MA, USA).

### 2.8. Membrane Potential Assay

Rhodamine 123, a fluorescent cationic dye, was employed as a probe to detect changes in membrane potential, reflecting the functional status of bacterial cell membranes. Rhodamine was dissolved in PBS to prepare a stock solution at a concentration of 1.0 mg/mL. Treated bacterial cells were then exposed to a working solution containing Rhodamine 123 at a final concentration of 2.0 μg/mL, followed by incubation in the dark at 37 °C for 30 min to ensure optimal dye uptake without photobleaching. The samples were subsequently transferred to a black 96-well plate to minimize background fluorescence and enhance detection sensitivity. Fluorescence intensity was measured using a fluorescence spectrophotometer (Infinite E Plex, Tecan Austria GmbH, Grodig, Austria), with excitation and emission wavelengths set at 480 nm and 530 nm, respectively, to accurately monitor the membrane potential changes [29].

### 2.9. Visualization of Membrane Integrity and Nucleic Acid Leakage

Confocal Laser Scanning Microscopy (CLSM) was employed to assess membrane integrity and intracellular DNA leakage following treatment. Treated bacterial cells were stained sequentially with two dyes, 20 μg/mL DAPI (4′,6-diamidino-2-phenylindole, Biofroxx, Germany), which binds to DNA and highlights the nucleoid region, and 20 μg/mL FM4-64 (N-(3-triethylammoniopropyl)-4-(6-(4-(diethylamino) phenyl) hexatrienyl) pyridinium dibromide), which specifically labels membrane components. The staining procedure included incubation with DAPI for 30 min to ensure thorough DNA labeling, followed by an additional 60 min incubation with FM4-64 at room temperature to visualize membrane structures. After staining, the bacterial suspensions were concentrated and mounted onto slides. Observations were performed using a CLSM equipped with a 63× oil immersion objective (ZEISS LSM 980, Carl Zeiss Microscopy GmbH, Jena, Germany), enabling high-resolution imaging of membrane integrity and structural alterations [30].

### 2.10. Statistical Analysis

All experiments were repeated three times. Data were statistically analyzed using Analysis of Variance (ANOVA), followed by Tukey’s post hoc test, with statistical significance defined as *p* < 0.05. The results were analyzed using SPSS software (version 25.0; IBM Corporation, Armonk, NY, USA), and GraphPad Prism (version 8.0.2; GraphPad Software, San Diego, CA, USA) was used for graphing.

## 3. Results and Discussion

### 3.1. Antibacterial Efficacy of Individual Organic Acids and Washing Sanitizers

MIC and MBC values of CA, TA, NaOCl, and PAA against *Salmonella* Typhimurium and *E. coli* O157:H7 were first determined, to establish a quantitative basis for evaluating their potential synergistic antibacterial effects. As shown in Table 1, the antibacterial efficacy against *Salmonella* Typhimurium and *E. coli* O157:H7 demonstrated a marked variation between organic acids and washing sanitizers.

It can be observed that among the two organic acids tested for disrupting the pathogens, TA exhibited the lowest MIC value of 3.0 mM against *E. coli* O157:H7, while CA showed the highest MIC value of 8.0 mM against *Salmonella* Typhimurium. For NaOCl, the MIC values were 300 ppm for *Salmonella* Typhimurium and 180 ppm for *E. coli* O157:H7, whereas the MIC values for PAA were 30 ppm and 25 ppm, respectively. Consistent with previous findings [31], the data also indicate that the MIC and MBC values of organic acids for *E. coli* O157:H7 were lower, showing that organic acids can effectively disrupt *E. coli* O157:H7 growth, while *Salmonella* demonstrates lower sensitivity to certain organic acids. For washing sanitizers, *Salmonella* Typhimurium also exhibited greater tolerance than *E. coli* O157:H7, especially to NaOCl. A study found that over 90% of *Salmonella* isolates have MICs greater than 256 ppm for NaOCl, indicating a high tolerance level, aligning with our results, where *Salmonella* Typhimurium displayed an MIC of 300 ppm [32]. This resistance may be associated with the activity of the *qacEA*1 gene, which encodes a chlorine-resistant efflux pump in *Salmonella.* Moreover, for both strains, the MBCs of NaOCl and PAA were equal to their MICs, indicating strong bactericidal activity rather than mere growth inhibition.

### 3.2. Synergistic Antibacterial Effects of CA-NaOCl and CA-PAA Combinations Against Salmonella Typhimurium and E. coli O157:H7

After determining the MICs and MBCs of individual agents, the synergistic antibacterial effects of various combinations against *Salmonella* Typhimurium and *E. coli* O157:H7 were investigated. Specifically, in this study, *Salmonella* Typhimurium treatments were designated as Combination 1 (CA-NaOCl) and Combination 2 (CA-PAA), while *E. coli* O157:H7 treatments were labeled Combination 3 (CA-NaOCl) and Combination 4 (CA-PAA), where “CA-NaOCl” and “CA-PAA” refer to combined treatments of the respective agents.

As summarized in Table 2, combinations of CA with NaOCl or PAA demonstrated the strongest synergy against *Salmonella* Typhimurium, with FICI values of 0.75 (CA-NaOCl) and 0.67 (CA-PAA). FICI is a well-established indicator for quantifying antibacterial synergy, reinforcing the validity of these findings. This is consistent with results reported by [33], where organic acid combinations yielded similar FICI values accompanied by strong synergistic effects against foodborne pathogens, thereby substantiating the rationale for acid-based synergistic interventions. In contrast, TA-PAA exhibited weaker synergy (FICI = 0.83), and TA-NaOCl was only additive (FICI = 1.00). A similar trend was observed for *E. coli*: O157:H7, CA-NaOCl and CA-PAA maintained synergistic effects (FICI = 0.75 and 0.90), while TA-NaOCl (FICI = 1.23) and TA-PAA (FICI = 1.47) showed indifference. Overall, these results indicate that CA-based combinations consistently outperformed those with TA in terms of synergistic antibacterial efficacy.

All combinations that yielded synergistic or near-synergistic effects occurred under highly acidic conditions (pH 1.72–1.98), suggesting that acidic environments contribute significantly to the enhanced bactericidal activity of oxidizing agents. However, pH alone did not fully explain the observed differences. Notably, TA-PAA exhibited only moderate synergy despite having the lowest pH (1.72), whereas CA-PAA showed the strongest effect at a slightly higher pH (1.82). These findings imply that factors beyond pH—such as acid type and bacterial strain-specific responses—play important roles in modulating the overall synergy. In this context, the superior performance of CA combinations suggests that intrinsic properties of CA—such as metal-chelating capacity, tricarboxylic structure, or enhanced membrane interactions—may contribute to its ability to potentiate oxidizing sanitizers.

These findings not only identified CA-NaOCl and CA-PAA as the most promising combinations based on FICI values, but also provided a solid foundation for subsequent dynamic validation using time-kill curves and practical application testing in a cherry tomato washing model.

### 3.3. Rapid Bactericidal Kinetics of Optimized CA-Based Combinations

Using the FICI method, CA-based combinations were found to exhibit stronger antibacterial activity than TA-based combinations against both foodborne pathogens. Four compound combinations showing the strongest synergistic bactericidal effects were further identified, each specifically targeting one of the two pathogens. Time-kill assays were plotted to validate the efficacy of the tested combinations.

**Figure 1:**
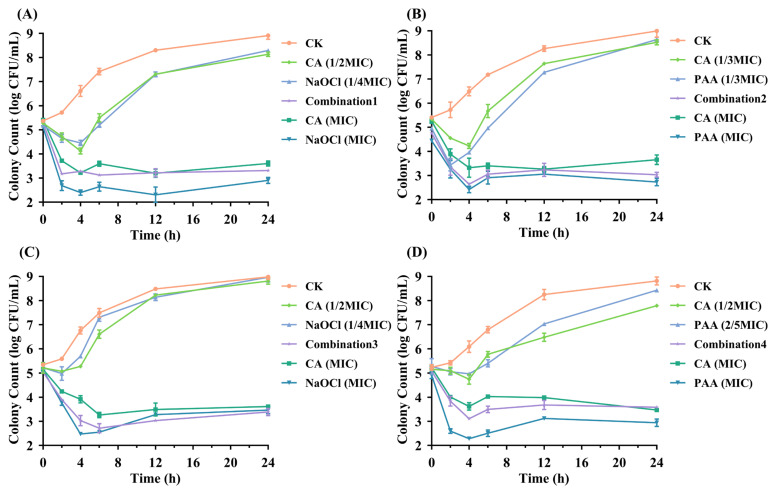
In vitro survival curves. CK (control group), CA (citric acid treatment), NaOCl (sodium hypochlorite treatment), Combination 1 (CA-NaOCl against *Salmonella* Typhimurium), Combination 2 (CA-PAA against *Salmonella* Typhimurium), Combination 3 (CA-NaOCl against *E. coli* O157:H7), and Combination 4 (CA-PAA against *E. coli* O157:H7) are indicated. (**A**–**D**) Time-kill curves of each bacterial strain for the indicated treatments. CFU, colony forming units. Statistical analysis was performed using two-way ANOVA followed by Tukey’s multiple comparisons test. Significant effects of treatment, time, and their interaction were observed (*p* < 0.05).

As shown in Figure 1, the results demonstrated that both CA combined with NaOCl and PAA effectively disrupted the growth of these pathogens compared to control and sub-MIC treatments. In the case of *Salmonella* Typhimurium, Combination 1 exhibited a pronounced antibacterial effect, achieving a maximum reduction of 2.16 log CFU/mL during the 24 h incubation period (Figure 1A). In contrast, CA (1/2 MIC) and NaOCl (1/4 MIC) alone exhibited little to no antibacterial activity, maintaining bacterial counts at 8.13 ± 0.03 log CFU/mL and 8.29 ± 0.06 log CFU/mL, respectively. Moreover, results from Combination 3 indicate that the antibacterial activity of the CA-NaOCl combination against *E. coli* O157:H7 was most pronounced after 12 h, even surpassing that of NaOCl alone (Figure 1C). These findings confirmed the synergistic effect of combination therapy, which achieved the antibacterial effect of single-agent MIC dose and improved bactericidal efficiency.

From the four sets of graphs, the combination treatments and the use of organic acids or washing sanitizers at MIC levels resulted in a rapid bacterial reduction within 2 h, demonstrating the rapid bactericidal ability of the combinations. This approach also confirmed a significant reduction in required washing sanitizer levels (approximately 2.5–4.0 times) and CA usage (2.0–3.0 times) compared to single agents. In contrast, CA, NaOCl and PAA at sub-MIC levels showed an initial reduction in pathogen counts within 2–4 h, followed by a gradual increase over time (ranging from 3.43 to 8.97 log CFU/mL).

Notably, the combination treatments exhibited greater stability in antibacterial effects compared to organic acids (MIC) or washing sanitizers (MIC). After CA (MIC) treatment, colony counts in three graphs (Figure 1A–C) slightly rebounded following their minimum levels, indicating the tolerance development in the bacteria [34]. As illustrated in Figure 1A,C, the resurgence of *Salmonella* Typhimurium and *E. coli* O157:H7 after 12 h of NaOCl (MIC) treatment may result from interactions between NaOCl and bacterial constituents (e.g., extracellular DNA or proteins), potentially reducing the residual concentration and antibacterial efficacy of NaOCl [35]. Overall, the time-kill curves visually illustrated the high efficiency of combination treatments, providing enhanced microbial safety in food while reducing washing sanitizer usage and cost.

### 3.4. Superior Bactericidal Efficacy of CA-NaOCl Combination Through Optimized Cherry Tomato Washing

To further investigate, cherry tomatoes were selected as a representative model for fresh produce. They were chosen because of their high consumption, susceptibility to microbial contamination, and surface characteristics that reflect those of many fresh fruits and vegetables. This practical model reflects real-world conditions and is relevant for improving microbial safety in both household and industrial produce sanitation. As shown in Table 3 and Table 4, washing contaminated tomatoes with water alone left residual pathogen levels of 5.09–5.22 log CFU/cm^2^, indicating that water alone cannot effectively remove microbial contaminants from fresh produce [23].

As shown in Table 3, the CA-NaOCl combination achieved a 6.01 log CFU/cm^2^ reduction relative to the initial inoculum on cherry tomato surfaces, and a 2.94 log CFU/cm^2^ reduction compared to water washing. In contrast, individual treatments with CA (1/2 MIC) and NaOCl (1/4 MIC) reduced pathogen counts by only 1.11–1.85 log CFU/cm^2^. These findings confirm that the combined treatment provided significantly enhanced antibacterial efficacy, demonstrating a synergistic effect in reducing foodborne pathogens on fresh produce. A previous study reported that the integration of pulsed light with a nisin–organic acid antimicrobial wash achieved a >5 log CFU/g reduction in *E. coli* O157:H7 [36], reaching the FDA’s threshold for pasteurization-level inactivation and indicating a strong synergistic effect. Additionally, the bactericidal effect of the CA-NaOCl combination was comparable to that of NaOCl at its MIC concentration (300 ppm), reducing NaOCl usage by four-fold while outperforming CA at its MIC concentration.

Table 4 showed that CA-PAA combination also demonstrated strong antibacterial effects in produce sanitation, reducing pathogen counts by 5.87 log CFU/cm^2^ compared to the inoculum level and slightly outperforming CA (MIC). This combination achieved equivalent or even higher antibacterial effects while reducing the usage of organic acids and polyacrylic acid by three times. Both treatment methods significantly reduced the required concentration of antibacterial agents, demonstrating valuable potential for practical application. Furthermore, it was encouraging to find that the CA-NaOCl combination achieved the most effective reduction in foodborne pathogens on cherry tomato surfaces while minimizing the use of food-grade antibacterials.

Additionally, the CA-NaOCl combination effectively reduced NaOCl concentration while maintaining strong pathogen inactivation, thereby minimizing potential concerns related to chlorine residues and byproduct formation. Therefore, given its superior antibacterial efficacy, practical feasibility, and cost-effectiveness, the CA-NaOCl combination was selected as the optimal formulation for further investigation. Moreover, mechanistic investigations were conducted to examine how the optimized CA-NaOCl combination disrupts membrane integrity, alters permeability, and impairs the metabolic functions of *Salmonella* Typhimurium, which was selected because, like *E. coli* O157:H7, it is a common Gram-negative bacterium with a similar cellular structure; however, *Salmonella enterica* has the broadest infection range among foodborne pathogens and exhibited higher resistance to the antimicrobial agents based on the MIC data, making it a representative model for mechanistic studies.

### 3.5. Extensive Morphological Damage Induced by CA-NaOCl Combination in Salmonella Typhimurium

Given the significant antibacterial activity of the CA-NaOCl combination (CA-NaOCl combination), formulated as 1/2 MIC CA + 1/4 MIC NaOCl, *Salmonella* Typhimurium was used as a model to visualize bacterial cell surface morphology changes following different treatments.

**Figure 2:**
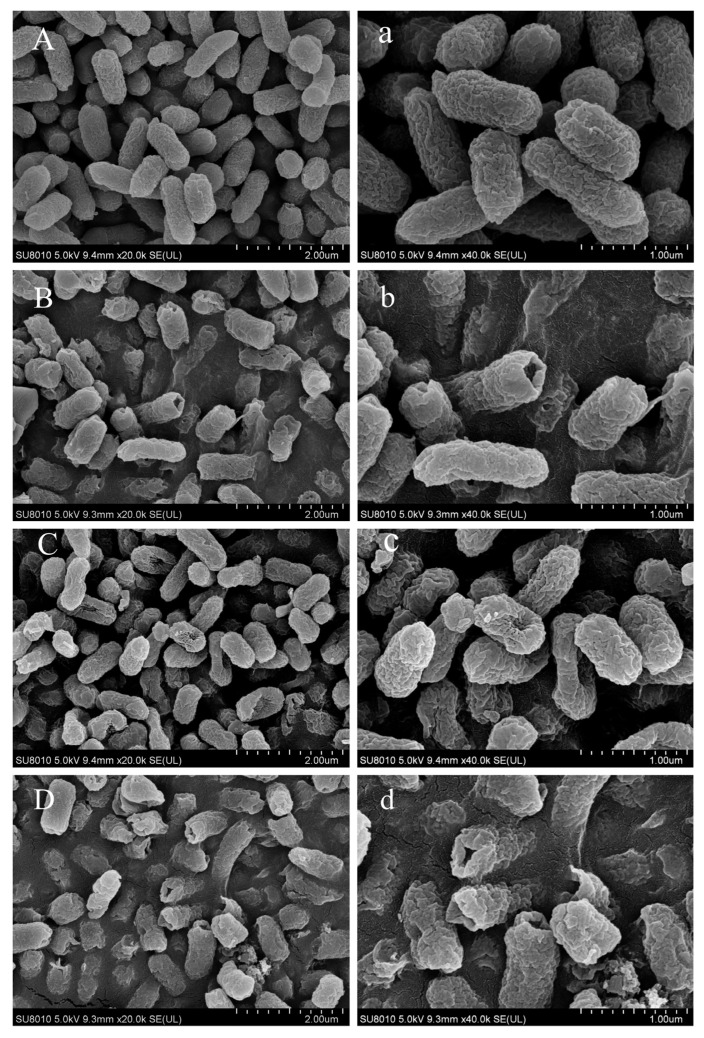
SEM images of Salmonella Typhimurium treated with different disinfectants. (**A**,**a**) Control group; (**B**,**b**) CA (1/2 MIC) treatment; (**C**,**c**) NaOCl (1/4 MIC) treatment; (**D**,**d**) CA-NaOCl combination (formulated as 1/2 MIC CA + 1/4 MIC NaOCl). Magnification for A, B, C, D = 20,000×, bar marker = 2.0 µm; magnification for a, b, c, d = 40,000×, bar marker = 1 µm.

As shown in Figure 2, control group cells displayed an oval shape with smooth surfaces. Exposure to CA (1/2 MIC) induced partial rupture or perforation of bacterial cell membranes, accompanied by leakage of intracellular components, suggesting that CA compromised both the cell wall and outer membrane integrity [37,38]. NaOCl (1/4 MIC) treatment resulted in concave deformation in the majority of cells, indicating that NaOCl, even at low concentrations, induced damage to the ultrastructure of *Salmonella* Typhimurium cells [39]. The effective chlorine component, HClO, reacts with amino acids and proteins, impairing enzymes on the cell membrane. Cells treated with the CA-NaOCl combination exhibited the most severe damage, with visible shrinkage and collapse, and most cells showed rupture or perforation, with content leakage (Figure 2). These results indicated a synergistic effect of NaOCl and CA on compromising the integrity of *Salmonella* Typhimurium cell walls and membranes, greatly enhancing the disinfectant effect of low-concentration NaOCl.

### 3.6. Membrane Disruption and Functional Impairment Induced by CA-NaOCl Combination

#### 3.6.1. Increased Leakage of Intracellular Contents Following Combination Treatment

Nucleic acids, proteins and other macromolecules are essential components of cells, participating in the regulation of microbial growth, reproduction, heredity, and metabolism. When the bacterial cell membrane is damaged, biomacromolecules leak into the extracellular environment. Therefore, the integrity of the cell membrane can be assessed by measuring the levels of nucleic acids and proteins in the supernatant.

**Figure 3:**
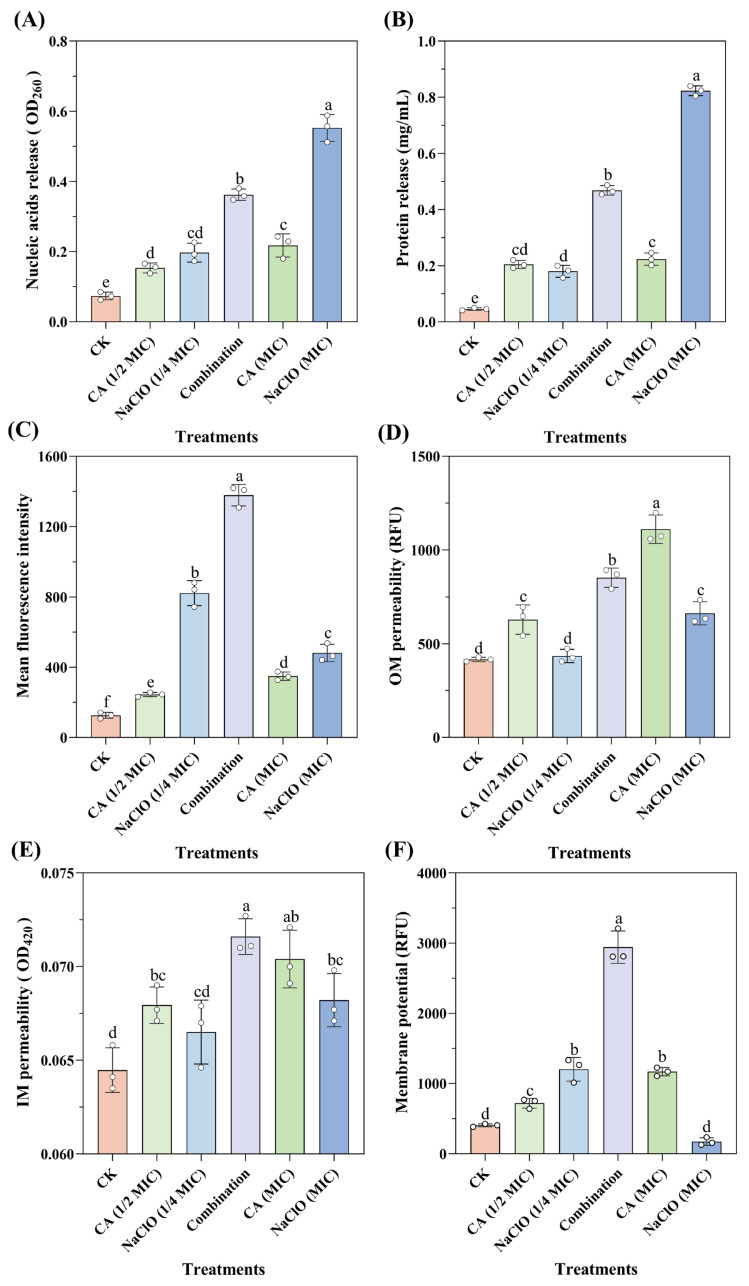
Membrane integrity and functional properties analysis. CK (control group), CA treatment, NaOCl treatment, and CA-NaOCl combination (formulated as 1/2 MIC CA + 1/4 MIC NaOCl) are indicated. (**A**) Determination of nucleic acid release of *Salmonella* Typhimurium by measuring the absorbance of the aqueous solutions surrounding the bacteria at OD_260_ upon treatments by CA, NaOCl, and CA-NaOCl combination. (**B**) Determination of protein release. (**C**) Integrity of the cell membrane measured by the uptake ability of PI after treatments. (**D**) Cell outer membrane (OM) permeability under different treatments. (**E**) Cell inner membrane (IM) permeability under different treatments. (**F**) Membrane potential changes upon different treatments. Error bars represent the standard deviation of three technical replicates (*n* = 3), with different lowercase letters indicating significant differences from each other (*p* < 0.05).

As shown in Figure 3A,B, higher concentrations of NaOCl caused increased leakage of intracellular nucleic acids and proteins. After treatment with NaOCl at the MIC level, protein leakage reached 0.823 mg/mL, indicating a significant disruption of intracellular macromolecules. NaOCl likely interacts with unsaturated fatty acids in phospholipids to form fatty acid chlorohydrins, thereby accelerating lipid peroxidation. In addition, NaOCl can rapidly react with amino (-NH_2_) and thiol (-NaOCl) groups, cleaving protein backbones and side chains, which in turn inactivates membrane-associated proteins and compromises membrane integrity [40].

In contrast, CA treatment at different concentrations showed limited effects on OD_260_ absorbance and protein leakage, consistent with previous observations [41]. This may be attributed to minimal pH changes from CA (1/2 MIC) to CA (MIC), resulting in similar levels of undissociated organic acid molecules and comparable impacts on membrane integrity. Notably, the CA-NaOCl combination induced significantly greater leakage of nucleic acids and proteins than either NaOCl (1/4 MIC) or CA (1/2 MIC) alone, with protein leakage nearly doubling compared to the CA (MIC) group. This enhanced leakage suggests more severe membrane disruption. A plausible explanation is that CA-induced membrane damage facilitated the intracellular penetration of NaOCl, which subsequently inflicted oxidative damage on nucleic acids and proteins, thereby amplifying its bactericidal efficacy through a synergistic mechanism.

#### 3.6.2. Compromised Membrane Integrity Detected by PI Staining

PI is a basic fluorescent dye with high affinity that cannot penetrate the cell membrane of viable cells but can enter cells with compromised membranes (i.e., dead cells or cells in late apoptosis), where it intercalates into DNA to form stable complexes, emitting red fluorescence [42]. Therefore, PI staining can be used to assess bacterial membrane integrity based on fluorescence intensity.

As shown in Figure 3C, the fluorescence value of the NaOCl (1/4 MIC) treatment was higher than that of the CA (MIC) treatment group, indicating that NaOCl treatment facilitated the permeation of PI into the cell membrane. However, high chloride levels significantly reduced the fluorescence signal of PI-stained DNA and RNA, as observed for fluorescence quenching after treatment with NaOCl (MIC). Specifically, at high chlorine concentrations (>3 mmol/L), nucleic acids were degraded before membrane integrity was compromised, resulting in false-negative results. Interestingly, one of the main bactericidal targets of NaOCl is the generation of ROS, which causes oxidative damage to DNA [43]. Additionally, the fluorescence value of the CA-NaOCl combination was approximately three times higher than that of the CA (MIC) treatment group, indicating that the presence of CA greatly facilitated NaOCl-induced disruption of the membrane integrity, as reflected by enhanced PI fluorescence. This aligns with the previous nucleic acid and protein leakage results, supporting the synergistic membrane-damaging effect.

#### 3.6.3. Synergistic Effect on Outer and Inner Membrane Permeability

The cell membrane, as a selectively permeable barrier, only allows the diffusion and transport of specific molecules or ions. Therefore, membrane permeability is one of the most crucial properties of the cell membrane. Reduced permeability can disrupt the exchange of substances between the cell and its environment or cause the leakage of low molecular weight compounds, which negatively affects bacterial physiological activity [44].

Firstly, the permeability of the outer membrane (OM) of *Salmonella* Typhimurium was measured using NPN staining under different treatments. As shown in Figure 3D, CA (MIC) treatment had a stronger effect on OM permeability than the NaOCl (MIC) treatment group, indicating that CA played a dominant role in influencing OM permeability. This is consistent with previous findings, where CA at millimolar concentrations was shown to be an effective permeabilizer, with its effects partially or almost completely eliminated by MgCl_2_, suggesting that the permeability effect may be due to the chelation of divalent cations on the cell membrane [45]. Organic acids may also interact with lipopolysaccharides on the cell membrane, destabilizing it and weakening the permeability barrier [18]. The CA-NaOCl combination also enhanced the impact on OM permeability compared to the NaOCl (MIC) treatment group, though not as strongly as the CA (MIC) treatment group.

The permeability of the inner membrane (IM) was assessed by measuring the release of cytoplasmic β-galactosidase. When the IM is disrupted, this intracellular enzyme leaks out and hydrolyzes the exogenous substrate o-nitrophenyl-β-D-galactopyranoside, producing a yellow product (ONP). As shown in Figure 3E, while the differences between CA (MIC) and the CA-NaOCl combination were not statistically significant, significant differences were evident between NaOCl (MIC) or CA (1/2 MIC) or NaOCl (1/4 MIC) and the CA-NaOCl combination. The CA-NaOCl combination exhibited the highest absorbance value of 0.072, indicating increased IM permeability, while the control group had the lowest value of 0.064.

CA treatment alone exhibited the most pronounced effect on both OM and IM permeability, as reflected by NPN and ONPG assays. This suggests that CA acted as a potent permeabilizing agent, disrupting membrane integrity and creating favorable conditions for NaOCl penetration into the cytoplasm. The enhanced intracellular leakage observed in the combination group may therefore result from CA-facilitated NaOCl entry, which amplifies its bactericidal efficacy. These findings support a synergistic mechanism in which CA initiates membrane damage, enabling NaOCl to exert more effective intracellular antibacterial action.

#### 3.6.4. Membrane Hyperpolarization Induced by CA-NaOCl Combination Treatment

The membrane potential arises from the difference in ion concentrations across the cell membrane, contributing to the proton motive force involved in ATP production, thereby influencing cellular metabolic activity. Changes in bacterial membrane potential serve as a key indicator of the mechanisms by which antibacterial agents act on membranes [46]. Hyperpolarization, similar to depolarization, is also an important indicator of bacterial activity. Rhodamine 123, a cationic fluorescent dye, can pass through the cell membrane and cell wall into the cytoplasm in a membrane potential-dependent manner, with its fluorescence intensity positively correlated with membrane potential.

According to the results depicted in Figure 3F, most cells in the different treatment groups exhibited membrane hyperpolarization following treatment. Studies have shown that treatment with organic acids leads to the accumulation of harmful anions in the cytoplasm, altering the intracellular pH and transmembrane transport of ions (especially H^+^ and K^+^) and resulting in membrane hyperpolarization. In an attempt to maintain electrochemical balance, cells undergo automatic regulation to hyperpolarize the membrane [47]. The fluorescence intensity in the CA-NaOCl combination was significantly higher than that in the CA (MIC) and the NaOCl (1/4 MIC) treatment group, indicating a synergistic effect of the CA-NaOCl combination on bacterial metabolic damage and intracellular proton accumulation. Additionally, high concentrations of NaOCl caused membrane depolarization, causing the extensive rupture and death of most bacteria, leading to a complete dissipation of membrane potential as the cells lose their ability to regulate it [48]. These findings suggest that CA-induced membrane hyperpolarization may facilitate NaOCl-induced intracellular proton accumulation and metabolic disruption, reinforcing the synergistic mechanism of action from the perspective of membrane potential disturbance.

### 3.7. Membrane Integrity Disruption and DNA Leakage Visualized by CLSM

In this study, CLSM was employed for the microscopic visualization of the membrane-targeted synergistic mechanism between CA and NaOCl, thereby strengthening the mechanistic interpretation of their combined antibacterial activity.

**Figure 4:**
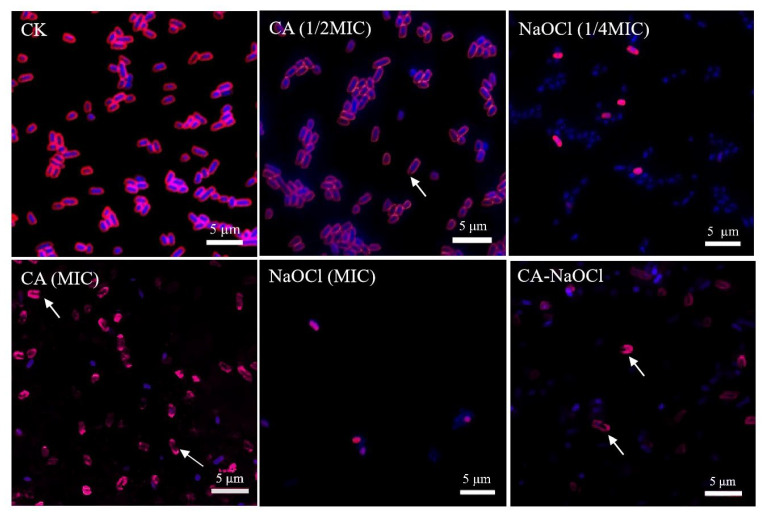
CLSM images of *Salmonella* Typhimurium under different treatments, stained with DAPI (blue fluorescence, specifically binding to intracellular double-stranded DNA) and FM4-64 (red fluorescence, selectively labeling the outer leaflet of intact phospholipid membranes). CK (control group), CA treatment, NaOCl treatment, and CA-NaOCl combination (formulated as 1/2 MIC CA + 1/4 MIC NaOCl) are indicated. The scale bar represents 5.0 µm. Arrows highlight nucleic acid leakage and membrane structural abnormalities.

As depicted in Figure 4, CA caused severe membrane damage even in the CA (1/2 MIC) treatment group, with fragment membrane fluorescence and leakage of intracellular nucleic acids. In the NaOCl-treated group, FM4-64 exhibited diminished and punctate fluorescence, which may be attributed to the oxidative action of NaOCl disrupting the phospholipid bilayer and membrane proteins. As FM4-64 requires an intact and tensioned membrane structure for stable intercalation, membrane shrinkage and disintegration likely prevented dye binding, resulting in diminished signal intensity. This interpretation is also supported by the wrinkled membrane morphology observed in SEM images. The observed weak fluorescence in the NaOCl treatment group may indicate the high antibacterial activity of NaOCl, which caused extensive cellular damage and compromised the fluorescence signal.

The fluorescence intensity of the combination formulation was weaker than that of the CA group, possibly because CA provided an optimal acidic environment for NaOCl action, enhancing the antibacterial efficacy of trace NaOCl [49]. The combination treatment caused near-complete loss of membrane structure and intracellular content, with no intact cellular morphology observed. Membrane integrity was severely compromised, and leaked nucleic acids were widely dispersed extracellularly, indicating extensive intracellular degradation. These findings suggest that the combination synergistically enhanced the bactericidal effects on membrane integrity and intracellular components, leading to membrane collapse. The low bacterial count in the combination group further reflected the potent bactericidal action of CA and NaOCl, resulting in few viable cells being available for analysis. From a pH perspective, the synergy was also validated. Under equivalent active chlorine conditions, hypochlorous acid (HClO) exhibits stronger bactericidal activity than hypochlorite ions (OCl^−^). Due to the negative charge of cell membranes, hypochlorite ions have limited penetration, whereas neutral hypochlorous acid readily enters the cell [50]. The addition of CA optimizes the acidic environment, promoting this highly effective cell disruption mechanism.

Together, the observed fluorescence patterns in *Salmonella* Typhimurium confirmed that the combination treatment induced more extensive membrane disruption and intracellular damage than either agent alone, visually reinforcing the proposed synergistic mechanism whereby the organic acid increases membrane permeability and facilitates NaOCl-mediated damage to membrane lipids and intracellular components.

## 4. Conclusions

In response to the challenge of maximizing food safety while minimizing chemical residues, organic acid-sanitizer treatments were optimized and a low-dose CA-NaOCl combination (1/2 MIC CA + 1/4 MIC NaOCl) was identified as a potent and practical strategy. This combination achieved a >6 log CFU/cm^2^ reduction in prevalent foodborne pathogens on cherry tomato surfaces, matching the efficacy of full-strength NaOCl while reducing chlorine usage four-fold. Mechanistic analyses revealed a membrane-targeted synergistic model, in which CA acts both as a chemical potentiator (acidifying the surface to favor hypochlorous acid, HClO) and a physical permeabilizer (destabilizing the outer membrane). This dual action facilitated HClO penetration, oxidative damage to lipids and proteins, and the leakage of macromolecules, leading to envelope collapse and intracellular dispersal as confirmed by electron microscopy and confocal imaging. Together, these findings establish an effective, low-residue disinfection protocol for fresh produce and provide a mechanistic blueprint for the rational design of next-generation sanitizers. Future work should evaluate its efficacy against diverse produce types, microbial biofilms, and a broader spectrum of foodborne pathogens.

## Figures and Tables

**Table 1:** MIC and MBC of organic acids and washing sanitizers against *Salmonella* Typhimurium and *E. coli* O157:H7.

Strain		CA	TA	NaOCl	PAA
*Salmonella* Typhimurium	MIC	8.0	6.0	300.0	30.0
	MBC	10.0	8.0	300.0	30.0
*E. coli* O157:H7	MIC	5.0	3.0	180.0	25.0
	MBC	6.5	3.5	180.0	25.0

Note: CA, citric acid (mM); TA, tartaric acid (mM); NaOCl, sodium hypochlorite (ppm, mg·L^−1^ available chlorine); PAA, peracetic acid (ppm). Values represent mean ± SD (*n* = 3). Concentration in ppm is expressed as mg·L^−1^.

**Table 2:** Combined antibacterial effect of organic acid with washing sanitizers against *Salmonella* Typhimurium and *E. coli* O157:H7.

Strain	Combination	FIC_O_	FIC_W_	FICI	Effect	pH
*Salmonella* Typhimurium	CA-NaOCl	1/2	1/4	0.75	synergism	1.90 ± 0.03
	CA-PAA	1/3	1/3	0.67	synergism	1.82 ± 0.02
	TA-NaOCl	1/3	2/3	1.00	commutative effect	2.68 ± 0.01
	TA-PAA	1/2	1/3	0.83	synergism	1.72 ± 0.06
*E. coli* O157:H7	CA-NaOCl	1/2	1/4	0.75	synergism	1.98 ± 0.02
	CA-PAA	1/2	2/5	0.90	synergism	1.88 ± 0.02
	TA-NaOCl	2/3	5/9	1.23	indifference	2.34 ± 0.04
	TA-PAA	2/3	4/5	1.47	indifference	1.79 ± 0.01

Note: The combined relationship is defined by the FICI interpretation model: FICI value < 1.0, Synergism; FICI value = 1, Commutative effect; 1 < FICI value ≤ 2, Indifference; FICI > 2, Antagonism.

**Table 3:** The effect of the CA-NaOCl combination (1/2 MIC CA + 1/4 MIC NaOCl) on the reduction level (log CFU/cm^2^) of the mixed Gram-negative pathogenic bacteria on the surface of cherry tomatoes. The average inoculum level on the surface of cherry tomatoes was 8.29 ± 0.06 log CFU/cm^2^. The control group was washed with purified water.

Treatment	Control	CA		NaOCl		CA-NaOCl Combination
		1/2 MIC	MIC	1/4 MIC	MIC	
Log reduction (log CFU/cm^2^)	3.07 ± 0.05 ^e^	4.18 ± 0.09 ^d^	5.73 ± 0.02 ^b^	4.92 ± 0.16 ^c^	6.11 ± 0.04 ^a^	6.01 ± 0.17 ^a^

Note: All values are presented as mean ± SD (*n* = 3). Different letters indicate significant differences (*p* < 0.05) within each row.

**Table 4:** The effect of the CA-PAA combination (1/3 MIC CA + 1/3 MIC PAA) on the reduction level (log CFU/cm^2^) of the mixed Gram-negative pathogenic bacteria on the surface of cherry tomatoes. The average inoculum level on the surface of cherry tomatoes was 8.29 ± 0.06 log CFU/cm^2^. The control group was washed with purified water.

Treatment	Control	CA		PAA		CA-PAA Combination
		1/3 MIC	MIC	1/3 MIC	MIC	
Log reduction (log CFU/cm^2^)	3.20 ± 0.02 ^c^	4.00 ± 0.29 ^b^	5.82 ± 0.01 ^a^	4.21 ± 0.05 ^b^	5.97 ± 0.13 ^a^	5.87 ± 0.01 ^a^

Note: All values are presented as mean ± SD (*n* = 3). Different letters indicate significant differences (*p* < 0.05) within each row.

## Data Availability

The original contributions presented in this study are included in the article. Further inquiries can be directed to the corresponding author.
